# Meldonium and human sport performance: a narrative review evaluating the evidence for ergogenic potential

**DOI:** 10.3389/fspor.2026.1822778

**Published:** 2026-06-09

**Authors:** Christer Malm, Michael Svensson

**Affiliations:** 1 Section for Sports Medicine, Department of Community Medicine and Rehabilitation, Umeå University, Umeå, Sweden; 2 Umeå School of Sport Sciences, Umeå University, Umeå, Sweden

**Keywords:** athletics, doping in sports, energy metabolism, fatty acid oxidation, glucose metabolism, metabolic modulators, mitochondria, sports medicine

## Abstract

Meldonium has attracted considerable attention in sports due to its suggested effects on energy metabolism and high-profile doping cases. This narrative review evaluates Meldonium's biochemical mechanisms, pharmacokinetics, safety profile, and the quality of evidence surrounding its use in athletes. While Western literature categorizes Meldonium as a metabolic modulator, Eastern literature often classifies it as an antihypoxic agent. By inhibiting carnitine biosynthesis, Meldonium shifts substrate utilization from fatty acid oxidation toward more oxygen-efficient glucose metabolism. However, despite its widespread use and inclusion on the World Anti-Doping Agency (WADA) prohibited list, there remains a profound lack of high-quality, randomized placebo-controlled trials demonstrating consistent ergogenic benefits in healthy athletes. Existing human performance studies exhibit a high risk of bias and methodological flaws. This review provides a critical appraisal of the current literature, highlighting the physiological trade-offs of Meldonium use and emphasizing the need for rigorous clinical trials to determine its genuine impact on athletic performance and long-term safety. This comprehensive review aims to clarify Meldonium's pharmacological profile, inform athlete health considerations, and guide future research into its legitimate clinical and sports applications.

## Introduction

While Western countries commonly refer to substances like Meldonium as “metabolic modulators,” in Eastern countries, they are frequently classified as “antihypoxic agents” (1). Antihypoxants can be broadly divided into four groups: inhibitors of fatty acid *β*-oxidation (e.g., Meldonium, trimetazidine, ranolazine), succinate-based drugs (e.g., Mexidol, Cytoflavin), natural components of the respiratory chain (cytochrome C, ubiquinone), and artificial redox systems (Hypoxen) (2).

The World Anti-Doping Agency (WADA) classified Meldonium as a prohibited substance effective January 1, 2016. The decision to move Meldonium from the Monitoring program to the Prohibited list was made “…*because of evidence of its use by athletes with the intention of enhancing performance*” (WADA 2016 Prohibited List, 16 September 2015), rather than robust scientific evidence demonstrating actual performance enhancement.

To date, scientific consensus on Meldonium’s ergogenic effects in humans remains inconclusive, prompting a need for a critical evaluation of the existing evidence hierarchy.

Meldonium, developed in 1974 by Ivars Kalvin at the Latvian Institute of Organic Synthesis, was initially intended to enhance the fertility and growth of livestock (3). The transition to human therapeutics came via *ex vivo* models by Chiba et al. (4) confirming the drug's baseline cardiovascular safety, in 1989, Dudko et al. (5) demonstrated that Meldonium exerted antianginal effects and successfully improved the physical working capacity of patients suffering from stable effort angina. Germane (6) showed Meldonium's benefits to positively affect the central nervous system. Rat models (7) demonstrated that Meldonium acts as a non-competitive inhibitor of ɣ-butyrobetaine hydroxylase, dramatically depleting tissue L-carnitine levels and forcing a compensatory shift in fatty acid oxidation. Together, this sequence of studies established the foundation for Meldonium's role as a safe metabolic modulator capable of protecting tissues during hypoxic or ischemic stress.

Meldonium is readily available (often over the counter without a prescription) in Russia and several other Eastern European and Central Asian nations. Meldonium is a medicinal product formally authorized within the European Union. Through the European Decentralized Procedure (DCP) and national registries, it is legally approved for use in selected EU Member States, including Latvia, Lithuania, and Poland. Outside the EU, it is widely registered for use in countries such as Russia, Ukraine, Georgia, Kazakhstan, Azerbaijan, Belarus, Uzbekistan, and Moldova, but not in the United States.

The drug's Latvian manufacturer, JSC Grindeks, reports that it primarily targets individuals with heart disease, and “*During increased physical activity, it restores the oxygen balance of tissue cells as well as activating the metabolic processes that result in lower requirements of oxygen consumption for energy production*”, and also that “*Meldonium cannot improve athletic performance, but it can stop tissue damage in the case of ischemia*” (https://grindeks.com/en/grindeks-meldonium-should-not-be-included-in-the-prohibited-list/).

Therefore, this narrative review aims to critically evaluate published scientific literature to determine whether Meldonium's unique metabolic mechanisms actually translate into documented performance-enhancing benefits for healthy athletes, or if its ergogenic reputation is largely unfounded.

## Methodological considerations

This manuscript is framed as a narrative review. Because of the broad scope required to synthesize Meldonium's historical context, pharmacological mechanisms, and varying international regulatory statuses, it does not employ formal systematic methodologies, such as a PRISMA flow diagram, strict inclusion/exclusion criteria, or standardized risk-of-bias assessment tools. However, to ensure scientific rigor and provide a meaningful evaluation of the drug's performance-enhancing potential, this review actively applies critical appraisal to the available human and animal studies. Throughout the manuscript, we critically evaluate the hierarchy and reliability of the evidence by specifically emphasizing methodological limitations, study design flaws, the presence or absence of placebo controls, and baseline population equality.

Because humans and animals have different metabolism, physiology and pharmacokinetics, animal studies are only briefly mentioned as results cannot be directly transferred to athletic performance.

## Methods

### Literature search and selection

This narrative review was constructed through a comprehensive search of peer-reviewed publications, authoritative texts, and official anti-doping reports related to Meldonium, metabolic modulation, and athletic performance. Databases searched include PubMed, Scopus, and Web of Science, focusing on articles published up to Feb 2026. Relevant papers were identified using Medical Subject Headings (MeSH) such as “Meldonium,” “Ergogenic Aids,” “Athletic Performance,” “Sports Medicine,” and “Energy Metabolism.” Reference lists of retrieved articles were screened for additional sources. In addition, available AI tools (ChatGPT, Perplexity, SciSpace, Gemini, NotebookLM) were used for in-depth search of the Internet, data extraction, translation and manuscript proof reading.

### Methodology and quality of evidence assessment

This narrative review incorporates literature across a hierarchy of evidence, ranging from animal models and small clinical cardiology trials to observational and interventional athletic studies. It is critical to note that the available data on Meldonium in healthy athletes currently carries a high risk of bias. Many existing studies lack robust placebo controls, feature imbalanced baseline cohorts, or are published in non-indexed journals. Consequently, the strength of any conclusions regarding Meldonium's performance-enhancing capabilities must be carefully weighed against the significant methodological limitations of the underlying source material.

### Eligibility and data synthesis

Studies included original human and animal research, systematic reviews, narrative reviews, and expert consensus statements. Inclusion criteria prioritized works that contributed to understanding Meldonium's mechanisms, effects, prevalence, clinical use, and controversy in sport. Data were synthesized narratively, highlighting major themes, findings, and gaps.

### Overview of meldonium

**Figure 1:**
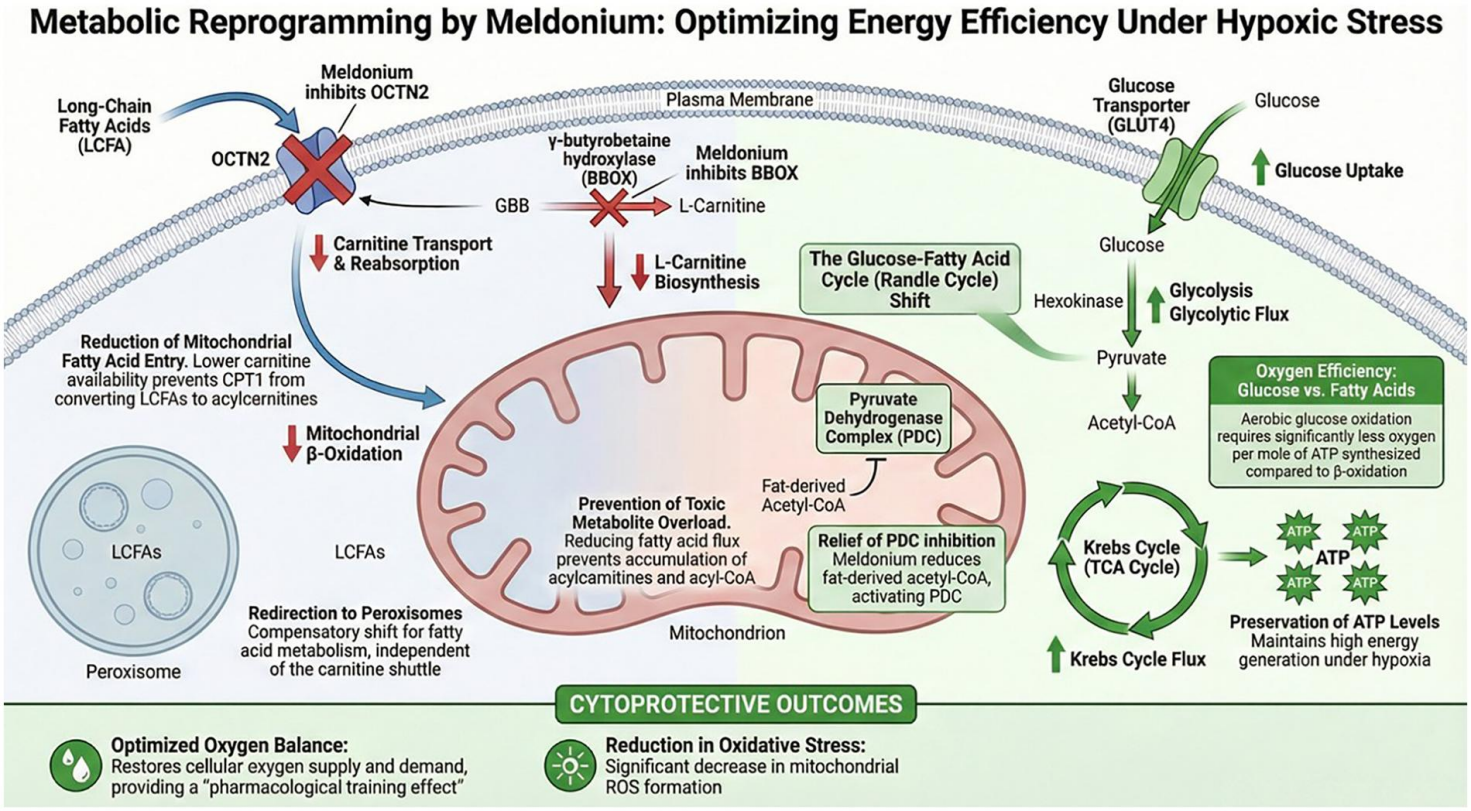
The correlation between fatty acid beta-oxidation, the krebs cycle, glucose utilization, and reduced oxygen consumption. Generated with NotebookLM.

Meldonium is a synthetic compound used in certain regions to manage ischemic conditions, including myocardial infarction (5, 8) and is believed to enhance exercise tolerance in patients with stable angina (9). Meldonium is commonly marketed under the brand name Mildronate® (also Mildronāts, MET-88, THP and Quaterine), though additional names have been employed. The proposed mechanism of action for improved physical performance involves inhibiting the final step in carnitine biosynthesis. Under oxygen-deficient conditions, there is a diminished oxygen supply and lower free carnitine levels, which reduces fatty acid metabolism but enhances glycolysis (Figure 1). This increases the efficiency of ATP production (10–12). Meldonium is currently registered for human use in some European countries and Russia to treat cardiovascular conditions, where it is available via online pharmacies, thus potentially misused in sport (13).

### Pharmacological physiology and mechanism of action

#### Pharmacological physiology and mechanism of action

Meldonium [3-(2,2,2-trimethylhydrazinium)propionate] is a structural analogue of gamma-butyrobetaine (GBB), an intermediate in the L-carnitine biosynthesis pathway. It inhibits gamma-butyrobetaine hydroxylase (*γ*-butyrobetaine dioxygenase), thereby reducing endogenous L-carnitine availability (7). Because L-carnitine is required for the mitochondrial import of long-chain fatty acids via the carnitine shuttle (CPT1–CACT–CPT2), lowering carnitine limits fatty-acid *β*-oxidation (Figure 1).

#### Carnitine depletion and OCTN2 inhibition

In addition to inhibiting carnitine synthesis, Meldonium inhibits the organic cation/carnitine transporter OCTN2 (SLC22A5), which mediates cellular uptake and renal reabsorption of carnitine (14). Animal data indicate that Meldonium depletes intramuscular L-carnitine, reduces tissue acylcarnitines, and suppresses mitochondrial *β*-oxidation in skeletal and cardiac muscle, shifting ATP production toward carbohydrate oxidation and glycolysis (15, 16). These changes are consistent with impaired uptake/retention of both L-carnitine and its precursor GBB (14, 16).

#### Substrate shift under hypoxia/ischemia

By constraining fatty-acid *β*-oxidation, Meldonium promotes a greater reliance on glucose-derived acetyl-CoA and oxaloacetate, supporting continued flux through glycolysis and the Krebs (TCA) cycle. Under conditions where oxygen availability becomes limiting (e.g., ischemia or possibly exercise-associated hypoxia), this metabolic shift optimizes cellular energy production. Specifically, carbohydrate oxidation is significantly more oxygen-efficient than fatty acid *β*-oxidation because less oxygen is required per molecule of ATP generated (17). By shifting substrate utilization toward glycolysis, Meldonium maximizes ATP yield per unit of available oxygen while simultaneously preventing the accumulation of potentially harmful lipid-derived intermediates under stress (17). Furthermore, Meldonium has been reported to influence upstream glucose handling, such as via the activation of hexokinase, further supporting enhanced carbohydrate utilization (15).

#### Downstream adaptations and enzyme-level considerations

Metabolic redirection with Meldonium has been associated with changes in transcriptional regulators linked to mitochondrial/peroxisomal remodeling (e.g., PGC-1*α*, PPAR*α*) (15). Importantly, Meldonium does not appear to directly inhibit key mitochondrial enzymes such as CPT1 or carnitine acetyltransferase *in vivo*, and inhibition of the carnitine/acylcarnitine translocase is relatively weak; instead, the dominant effect is mediated through altered carnitine availability (15). Collectively, these mechanisms underpin its proposed therapeutic role in cardiovascular disease (e.g., angina, myocardial infarction) (18).

### Biotransformation and potential relevance of metabolites

Most scientific publications focus on the parent compound [3-(2,2,2-trimethylhydrazinium) propionate], but biotransformation products may also be relevant. Meldonium has been described as metabolized via 3-hydroxypropionic acid with formation of succinic acid (19, 20). Succinic acid is a central intermediate of the Krebs cycle and is also a key component of several antihypoxic drug concepts (21).

### Effects on oxidative stress and inflammation-linked mitochondrial dysfunction

Hypoxia and intense exertion can promote inflammatory signaling and oxidative stress, contributing to mitochondrial dysfunction; mitochondrial damage can further amplify inflammation via ROS generation and impaired ATP production (22–24). Across animal models (notably LPS-induced inflammation/sepsis), Meldonium has been reported to reduce oxidative damage markers and inflammatory/apoptotic signaling while increasing antioxidant enzyme activity, consistent with cytoprotective and anti-inflammatory effects (15). Related protective effects have also been described in models of traumatic brain injury and other inflammatory cardiac stress settings (12, 25).

### Carnitine transport, homeostasis, and substrate selection

Meldonium not only inhibits GBB hydroxylase but also blocks the high-affinity organic cation/carnitine transporter OCTN2 (SLC22A5), which mediates cellular uptake and renal reabsorption of carnitine (14). Inhibition of OCTN2 reduces intracellular L-carnitine and its precursor gamma-butyrobetaine, suppresses mitochondrial transport of long-chain fatty acids, and lowers tissue acylcarnitine levels (14–16).

Animal studies in skeletal and cardiac muscle show that this disturbance of carnitine homeostasis diminishes fatty-acid *β*-oxidation and increases reliance on carbohydrate oxidation for ATP production, especially under hypoxic or ischemic conditions (15–17). The substrate shift is accompanied by changes in transcriptional regulators such as PGC-1*α* and PPAR*α*, consistent with mitochondrial and peroxisomal remodeling (15). Meldonium does not appear to directly inhibit CPT1 or carnitine acetyltransferase *in vivo*, and its inhibition of the carnitine/acylcarnitine translocase is comparatively weak; rather, the dominant effect is mediated through altered carnitine availability (15). Together, these mechanisms support its proposed use as a metabolic therapy in cardiovascular disease, including angina and myocardial infarction (18).

### Cytoprotective, vascular, and mitochondrial effects

Meldonium has been reported to exert vasodilatory effects, likely via nitric-oxide–related pathways, with potential implications for blood flow and oxygen delivery during exertion (26). Experimental work describes neuroprotective, antioxidant, anti-inflammatory, and anti-apoptotic actions across models of cerebral ischemia, ocular ischemic syndrome, alcohol withdrawal, and other CNS disorders (17, 22, 27–29).

Hypoxia and intense exercise promote inflammatory activation and oxidative stress that contribute to mitochondrial dysfunction, often via disturbed fusion–fission balance and accumulation of damaged mitochondria/mtDNA (22–24). Across multiple animal models (e.g., LPS-induced inflammation and sepsis), Meldonium reduced oxidative damage, attenuated inflammatory and apoptotic signaling, and increased activities of antioxidant enzymes such as Cu/Zn-SOD, Mn-SOD, catalase, and glutathione peroxidase (15). Similar protective effects have been reported in pulmonary-hypertension–related right-heart failure, cytokine-mediated cardiac dysfunction (including COVID-19 settings), and traumatic brain injury (12, 15, 25).

Mitochondrial remodeling in response to Meldonium is not necessarily benign: increased mitochondrial turnover or exercise-induced stress can elevate ROS and mtDNA vulnerability, particularly with impaired antioxidant defenses or DNA repair (12, 30, 31). Nevertheless, in a forced-swimming mouse model, Meldonium reduced exercise-induced mitochondrial dysfunction, oxidative damage, and apoptosis in cardiac tissue, consistent with cardioprotective potential (12).

In athletes, prolonged high-intensity exertion may lead to “athletic heart syndrome,” characterized by cardiac hypertrophy, inflammation, oxidative stress, mitochondrial dysfunction, and accumulation of acylcarnitines with disturbed substrate oxidation (22, 23). By inhibiting mitochondrial *β*-oxidation and activating glucose-metabolic pathways (e.g., 6-phosphofructokinase and pyruvate dehydrogenase), Meldonium shifts ATP production from lipids toward carbohydrates during exercise, which may reduce oxidative stress and improve metabolic flexibility (15). These properties underpin proposed cardioprotective effects in athletes and interest in Meldonium as a performance-supportive supplement, while also raising concerns about its ergogenic misuse (32).

Preclinical performance data are mixed. In a rat forced-swimming model of mild organophosphorus intoxication, Meldonium (60 mg/kg) produced a smooth, sustained increase in endurance and maximal swimming time, but was less effective than hypoxen and octodrine, and combined administration did not show synergistic effects (33).

### Metabolic modulators and clinical applications

Meldonium is often grouped with metabolic modulators/antihypoxic agents that optimize energy utilization by shifting substrate selection and improving efficiency, including inhibitors of fatty-acid *β*-oxidation (e.g., trimetazidine, ranolazine), succinate-based drugs (Mexidol, Cytoflavin), respiratory-chain components (cytochrome C, ubiquinone), and artificial redox systems (Hypoxen) (21). Preclinical models of cardiovascular, neurological, and pulmonary disease generally show beneficial effects on tissue hypoxia, oxidative stress, and functional outcomes (12, 13, 15, 34–36). In patients with exertional angina, the addition of Meldonium to standard therapy has been associated with improved clinical response (37).

### Sports and regulatory context

Because Meldonium shifts substrate utilization from fatty-acid oxidation toward carbohydrate metabolism, it was first placed under monitoring and then, in 2016, added to the World Anti-Doping Agency Prohibited List under “S.4 Hormone and Metabolic Modulators”. In high-intensity exercise, this metabolic shift is hypothesized to increase energetic efficiency, reduce lactate accumulation, delay fatigue, and accelerate post-exercise recovery, which helps explain its attraction in elite sport (38, 39). If Meldonium inhibits fat oxidation and shifts metabolism toward glucose and glycogen breakdown, the resulting increase in glycolytic flux and pyruvate formation will automatically raise lactate production, because lactate dehydrogenase has a very high affinity for pyruvate once its concentration rises, regardless of mitochondrial density or full oxygen availability. This prediction still needs to be demonstrated experimentally.

Also, persistent uncertainty regarding long-term safety, limited high-quality human data in athletes, and its status as a banned substance continue to raise ethical and regulatory concerns (32).

### Human physical performance: mechanistic rationale vs. evidence

Since its inclusion on the WADA Prohibited List in 2016, Meldonium has attracted substantial attention as a potential performance-enhancing substance (9–13, 40). Nevertheless, direct evidence for ergogenic effects in healthy athletes is limited and methodologically weak (20).

### Mechanistic cardio-protective rationale

During intense or prolonged exertion, elite athletes may develop cardiac remodeling, transient myocardial hypoxia, oxidative stress, and accumulation of acylcarnitines, factors that can compromise function and promote arrhythmias (15, 22, 23). Meldonium is hypothesized to counter these changes by reducing carnitine-dependent fatty-acid *β*-oxidation, promoting glucose oxidation (e.g., via pyruvate dehydrogenase and hexokinase), and thereby improving myocardial energy efficiency under hypoxic conditions (11, 15).

Experimental work suggests modulation of PPAR-*δ*/PGC-1*α*–linked pathways, suppression of mitochondrial ROS, and improved recovery of cardiac function after ischemic or metabolic insult (15, 18). These findings are largely derived from animal studies and cell models; direct confirmation in human myocardium or skeletal muscle remains limited. In this context, previous reports of impaired performance with coenzyme Q10 supplementation illustrate that alterations of mitochondrial redox balance can be detrimental to high-intensity performance, underscoring the need for caution when extrapolating mechanistic benefits to athletes (41–43).

### Safety assessment and toxicity

In Russian and Eastern European literature, Meldonium is generally described as a low-toxicity compound (20, 39, 40). However, comprehensive long-term safety studies conducted according to contemporary international toxicology standards are scarce, particularly for healthy populations and athletes (44–46).

From a chemical standpoint, Meldonium's trimethylhydrazinium moiety is a potential toxicophore. Hydrazine-containing functional groups have been associated with hepatotoxicity and possible genotoxicity (47–49). While several hydrazine-based drugs (e.g., phenelzine) have been withdrawn, others such as hydralazine and isoniazid remain in use but require monitoring for drug-induced liver injury (36, 47, 50).

Hydralazine is a direct arteriole vasodilator with several critical indications: it is a primary intravenous option for hypertensive emergencies and severe pregnancy-related hypertension (as it effectively maintains blood flow to the uterus and fetus), a secondary option for resistant hypertension, and, when combined with isosorbide dinitrate, a mortality-reducing adjunctive therapy for severe heart failure (HFrEF), particularly in African-American patients. Although hydralazine and its derivatives exhibit broad therapeutic efficacy, with repositioning potential ranging from cardiovascular care to oncology (51), its clinical use requires careful management. To counteract compensatory side effects like rapid heart rate (tachycardia) and fluid retention, it is almost always prescribed alongside a beta-blocker and a diuretic. Furthermore, it carries a dose-dependent risk of hepatotoxicity and drug-induced lupus erythematosus. This risk for lupus-like syndromes is notably higher in individuals with a “slow acetylator” genetic variation, necessitating routine monitoring of complete blood counts and antinuclear antibody titers (52).

Consequently, while current data support the short-term tolerability of Meldonium, its long-term hepatic and mitochondrial safety profile, (especially in healthy athletes) requires more rigorous evaluation (20, 46).

### Athletic implications and controversies

While regular moderate exercise is generally cardioprotective, chronic high-intensity training may promote irreversible mitochondrial dysfunction, impaired ATP synthesis, and systemic inflammation (25, 31, 53). Meldonium's reported ability to reduce lactate production, limit ischemic injury, and enhance myocardial recovery has been interpreted as offering a potential performance advantage (32, 38, 39).

Such a mechanism seems unlikely to add any major benefit in healthy, highly trained athletes, who already display near-maximal PDH activation and finely tuned regulation of glycolytic flux under heavy exercise.

Some data suggest improved blood flow and tissue perfusion, potentially via nitric-oxide–mediated vasodilation, although effects on skeletal-muscle angiogenesis remain speculative (18, 26, 54).

Concerns have also been raised about safety in specific stress settings, including increased mortality in certain models of septic shock and other severe inflammatory states (55). Overall, the balance between potential cardiometabolic protection and the risk of conferring an unfair advantage—against a backdrop of incomplete long-term safety data—continues to drive debate in sports medicine and anti-doping policy (32).

### Animal studies of physical performance

Animal data supports Meldonium's therapeutic role in preventing cell damage and preserving function during extreme hypoxic (17) or ischemic stress (18, 56). However, it does not provide consistent or robust evidence that it improves maximal physical performance in healthy, uncompromised animals. While some long-term studies in healthy rodent models have found negligible effects on physical endurance, alongside adverse effects on gut microbiota and behavioral deviations (57), other highly specific models have demonstrated ergogenic potential.

**Figure 2:**
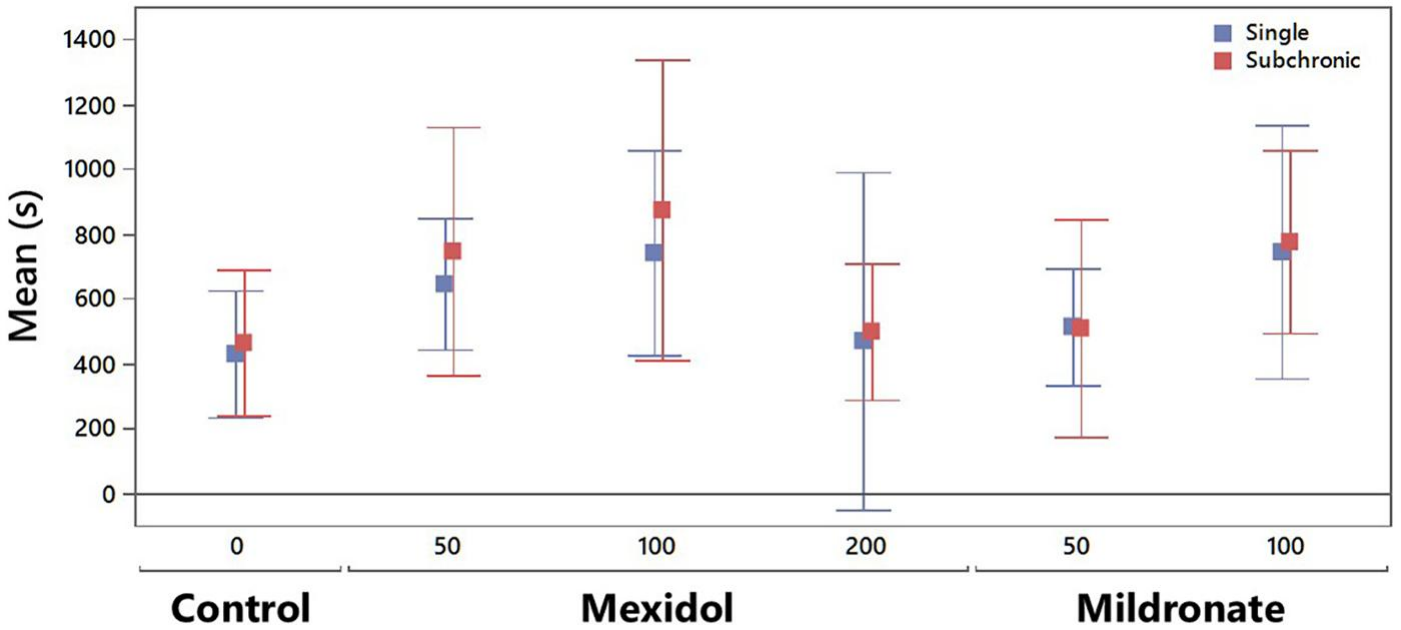
Effect of single and subchronic administration of mexidol and mildronate on physical performance in mice. The graph displays the mean swimming duration to exhaustion (in seconds) for mice carrying a load equal to 8% of their body weight. Data points represent the mean swimming times following a single intraperitoneal administration (blue squares) and subchronic 5-day administration (red squares) across the control group and varying dosage groups (50, 100, and 200 mg·kg⁻^1^). Error bars represent standard deviation (SD), illustrating the high degree of individual variability in physical endurance within the cohorts. Data extracted and calculated from Voronina et al. (2017).

For example, a 2017 study by Voronina et al. (58) utilizing a weighted forced-swimming test in healthy mice found that administration significantly increased time to exhaustion by up to 73% for Meldonium and 88% for Mexidol, compared to controls (Figure 2). However, this performance enhancement was strictly dose-dependent. It was only observed at 100 mg·kg⁻^1^, while lower doses (50 mg·kg⁻^1^) yielded no statistically significant endurance benefits. Furthermore, increasing the dose of Mexidol to 200 mg·kg⁻^1^ failed to improve swimming performance, suggesting a ceiling effect or over-dosing.

**Figure 3:**
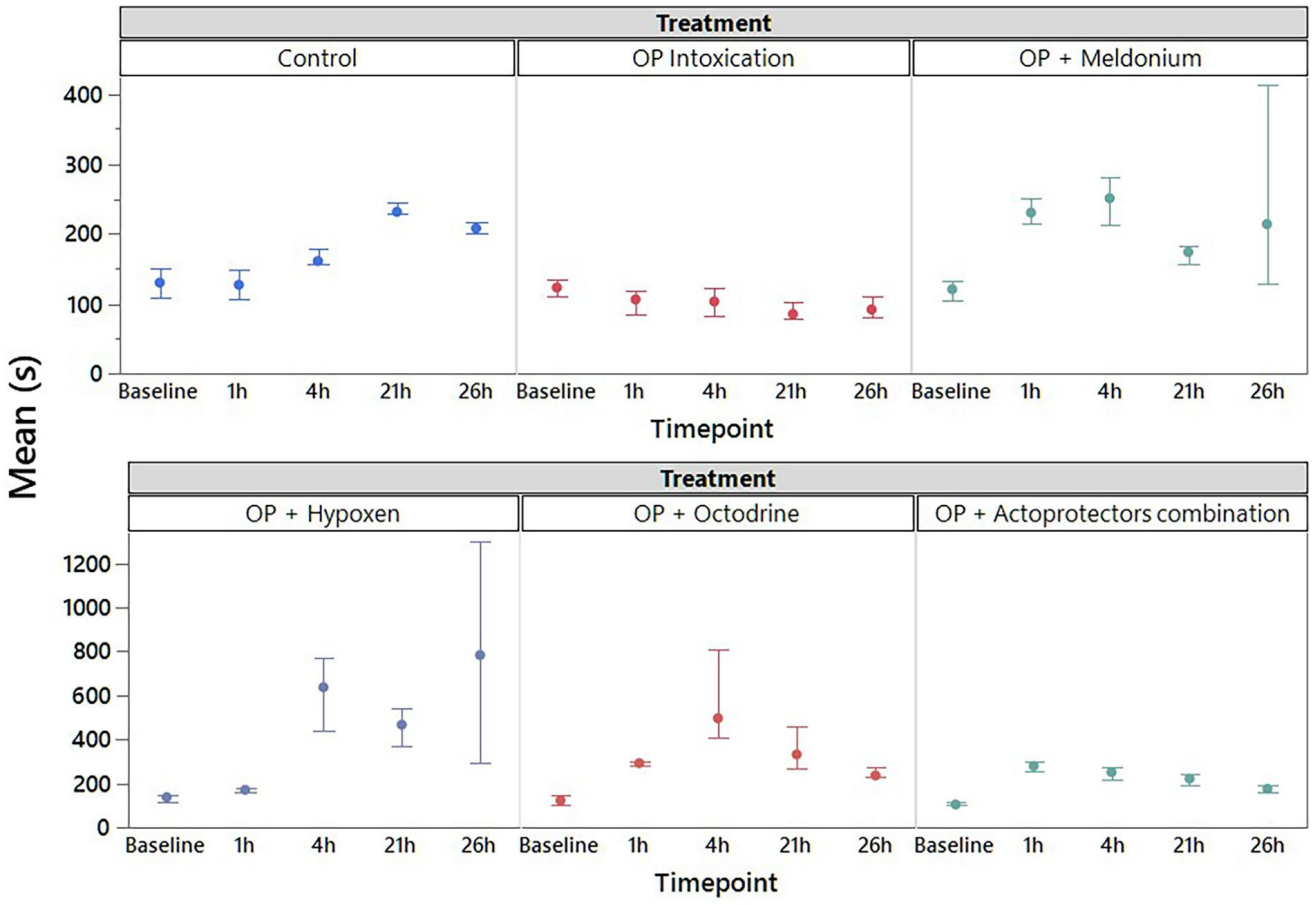
Maximum swimming duration (seconds) in the weighted forced-swimming test at baseline (day before) and 1, 4, 21, and 26 h after treatment in six rat groups: control; organophosphorus compound (OP) intoxication; OP + meldonium; OP + hypoxen; OP + octodrine; OP + actoprotectors combination. Mean with interquartile range (Q25–Q75). Data extracted and calculated from Mikshta et al. (2023). Cardiopulmonary exercise testing and recovery outcomes (mean ± SD) at baseline, Day 7, and Day 15 during mildronate (meldonium; red) vs. placebo (green): VO₂_max_ (mL·min⁻^1^·kg⁻^1^), VO₂ at anaerobic threshold (mL·min⁻^1^·kg⁻^1^), RER, VE/VO₂, O₂ pulse (mL·beat⁻^1^), and lactate at 5-min recovery (mmol·L⁻^1^). Data extracted and calculated from Goloborodko and Razinkin (2021).

When evaluated in pathological models, such as rats suffering from mild organophosphorus intoxication, Meldonium's effects were present but comparatively weak (33). Mikshta et al. demonstrated that while organophosphorus exposure reduced swimming time by ∼28%, single oral doses of actoprotectors (Meldonium, Octodrine, Hypoxen) helped preserve or increase endurance. However, when ranked by their effect on maximal loaded swimming time, Meldonium was the least effective (Meldonium < Octodrine < Hypoxen). A combination of all three drugs did not produce synergistic benefits; the combined effect was comparable to Meldonium alone, and inferior in magnitude and duration to Hypoxen (Figure 3).

Ultimately, while these animal models provide a mechanistic rationale for delayed exhaustion under extreme, unnatural physical stress (e.g., weighted swimming to the point of drowning), it remains highly speculative to extrapolate these rodent findings to the complex, voluntary physical performance of healthy elite human athletes.

### Human studies of performance and mood

**Figure 4:**
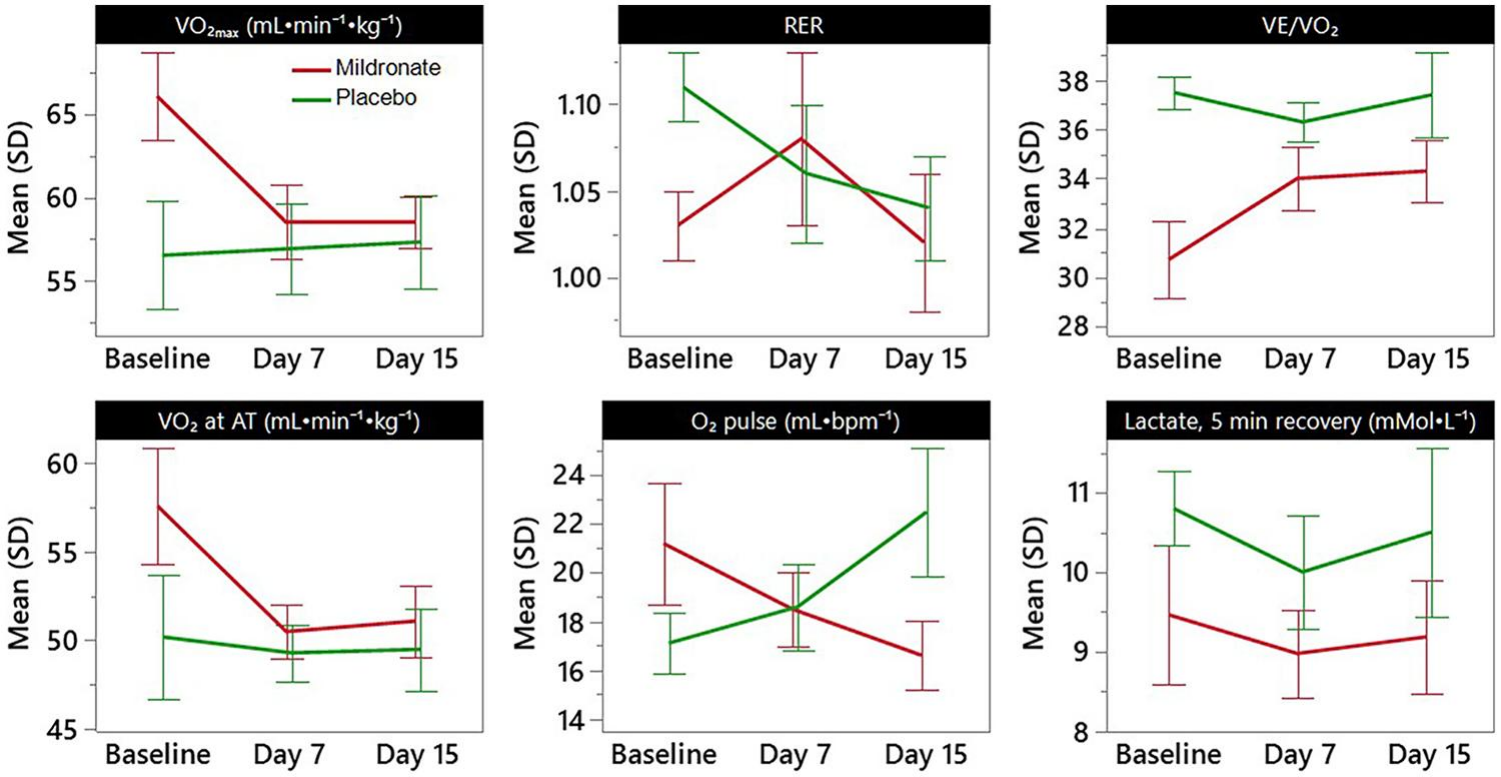
Cardiopulmonary exercise testing and recovery outcomes (mean ± SD) at baseline, Day 7, and Day 15 during Mildronate (Meldonium; red) versus placebo (green): VO₂max (mL·min⁻¹·kg⁻¹), VO₂ at anaerobic threshold (mL·min⁻¹·kg⁻¹), RER, VE/VO₂, O₂ pulse (mL·beat⁻¹), and lactate at 5-min recovery (mmol·L⁻¹). Data extracted and calculated from Goloborodko and Razinkin (2021).

The only placebo-controlled trial evaluating Meldonium in healthy athletes is a Russian-language study by Goloborodko et al. (2021), which examined Mildronate in 20 elite biathletes and cross-country skiers (10 men, 10 women; mean age 20.1 ± 0.6 years) over 15 days (59). While the original authors concluded that the drug improved “*functional readiness*”, a critical reanalysis of their published raw data reveals major methodological weaknesses. Most notably, allocation was performance-based rather than randomized. This resulted in severe baseline inequalities, with the Mildronate group exhibiting a mean VO₂_max_ of ∼66 mL·min⁻^1^·kg⁻^1^ compared to ∼56 mL·min⁻^1^·kg⁻^1^ in the placebo group (≈17% higher). This strongly suggests a sex imbalance (more men assigned to the Mildronate arm (but not explicitly stated in the publication) that precludes valid between-group comparisons (Figure 4).

**Figure 5:**
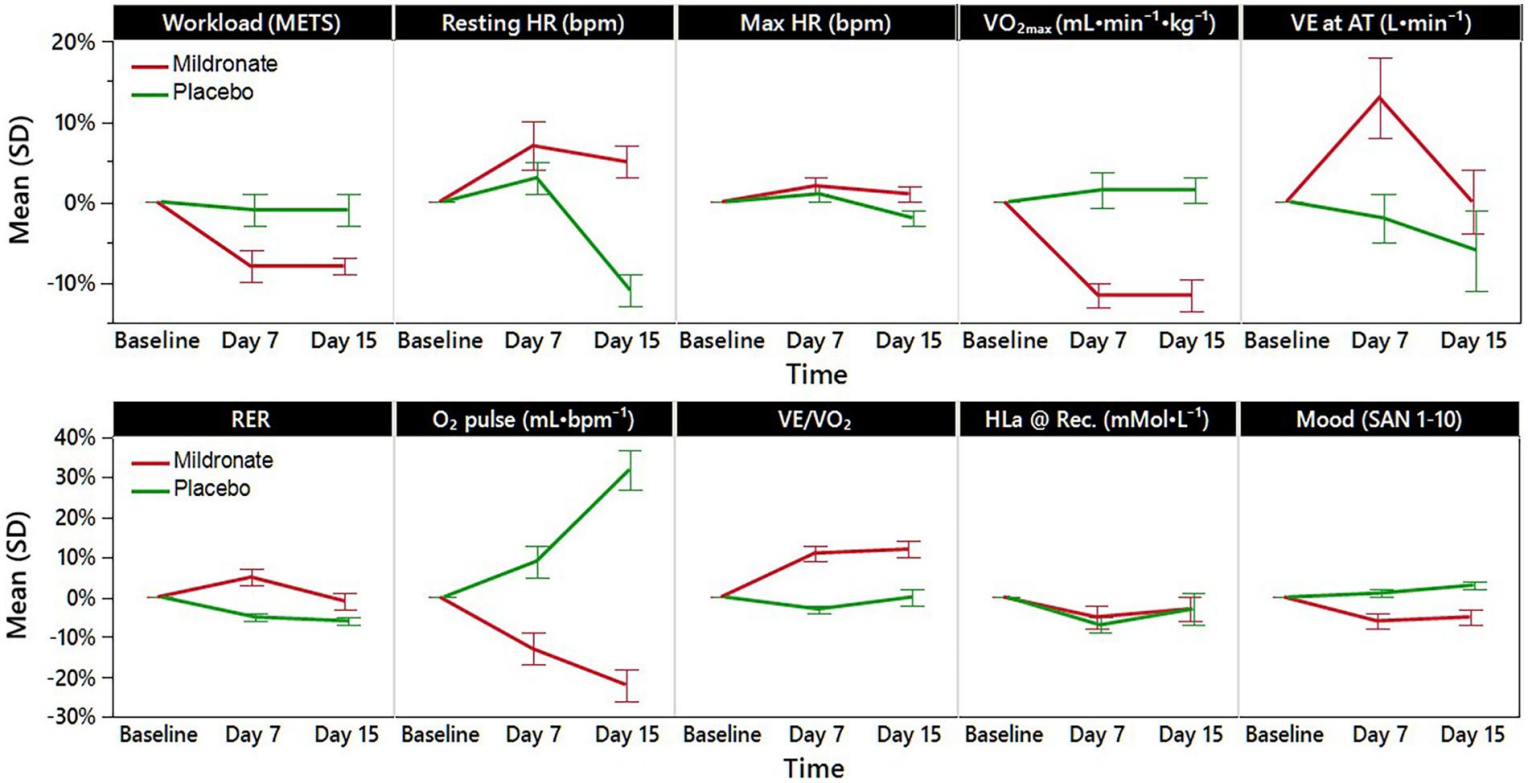
Percent change from baseline (mean ± SD) in workload (METs), resting heart rate, maximal heart rate, VO₂max, and ventilation at anaerobic threshold (VE at AT) across baseline, Day 7, and Day 15 during Mildronate (Meldonium; red) versus placebo (green). Data extracted and calculated from Goloborodko and Razinkin (2021).

Despite these design flaws, the tabulated data contradicts a clear ergogenic effect (Figure 5). Over the 15-day intervention, the Mildronate group showed a transient decline in VO₂_max_ and total workload, alongside an increased respiratory exchange ratio (RER), a finding consistent with a metabolic shift toward carbohydrate oxidation, but not with enhanced maximal performance (59). Furthermore, while group differences in heart-rate, ventilatory, and O₂-pulse responses were observed, the placebo group exhibited physiologically implausible changes (e.g., simultaneous reductions in resting and maximal HR), raising serious concerns regarding measurement reliability.

**Figure 6:**
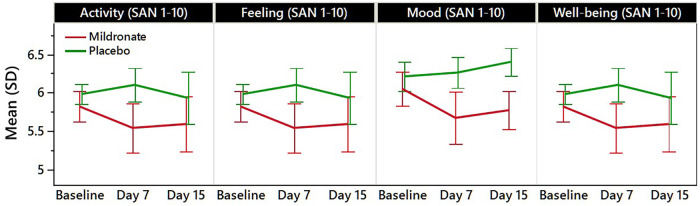
Changes in self-rated functional state on the SAN scale (1–10) during Mildronate (Meldonium) versus placebo. Mean (SD) scores are shown at baseline, Day 7, and Day 15 for the SAN subscales Activity, Feeling, Mood, and Well-being. Mildronate is shown in red and placebo in green. Data extracted and calculated from Goloborodko and Razinkin (2021).

Psychologically, the data also points toward potential adverse effects (Figure 6). On the SAN scale, subjective mood scores declined significantly by Day 15 in the Mildronate group compared with the placebo group (59). Additionally, the 15-day intervention period itself is problematic. Data on antihypoxic drugs, including succinate-based agents, suggest that meaningful ergogenic effects, as well as mitochondrial adaptations, typically require at least 14–21 days of continuous administration (21). Thus, the Goloborodko study may have been too short to capture any stable adaptations.

**Figure 7:**
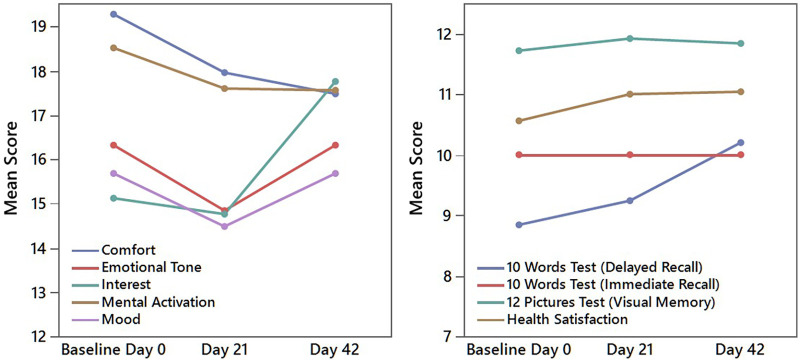
Changes in psychoemotional status and cognitive/mental performance measures during a 21-day course of Mildronate® (Meldonium) in 25 male youth ice hockey players, assessed at baseline (Day 0), Day 21, and Day 42. **Left panel:** Mean scores for psychoemotional-state domains (comfort, emotional tone, interest, mental activation, and mood) assessed using the Kurgansky method. **Right panel:** Mean scores for health satisfaction and psychophysiological tests of memory, attention, and mental performance, including the 10-word test (immediate and delayed recall), the “12 pictures” visual memory test, the Schulte–Platonov attention test, and the Health Satisfaction visual-analog scale. Values are group means derived from the study's tabulated results; the original article primarily reports means and, where applicable, min/max or categorical distributions rather than full dispersion measures for all outcomes. Data extracted and calculated from Chainikov et al. (2015).

Because athletic studies are so rare, an uncontrolled trial by Chainikov et al. (2015) in 25 male youth ice-hockey players (500 mg Mildronate twice daily for 21 days, followed for 42 days) is frequently cited (60). The authors originally reported improvements in mental performance, attention, memory, and psycho-emotional status using a battery of Russian neuropsychological tests (e.g., Luria 10-Word Test, 12 Pictures, Schulte–Platonov tables, Anfimov correction test, Kurgansky scale). However, these instruments have limited international validation, and the study critically lacked a placebo or control group and proper statistical analysis. While three parameters had higher mean values at Day 42, two decreased and three remained unchanged (Figure 7). While the authors recommended Mildronate to support psycho-emotional status, the proportion of players rating a state of “*high comfort*” actually fell from 12% at baseline to 0% at Day 42.

Taken together, available human data *suggests* that Meldonium can alter cardiopulmonary and psychological responses to exercise in trained athletes, but there is no robust evidence that it improves maximal aerobic performance (Table 1). In fact, critical re-evaluation points toward neutral or even adverse effects on VO₂_max_ and mood, despite plausible mechanistic arguments and cytoprotective findings in disease and injury models (59). These findings reinforce that mechanistic and animal data cannot be directly extrapolated to ergogenic benefits in healthy elite athletes.

**Table 1:** Summary of human studies on the effects of meldonium on physical performance.

Study	Study Design	Population	Outcomes	Limitations
Goloborodko et al. (2021)	Non-randomized, placebo-controlled trial	20 elite ski athletes (biathlon, cross-country skiing) 10 men, 10 women; mean age 20.1 ± 0.6 years	The placebo group reported the greatest number of positive subjective changes (mood, sleep, physical performance). The Mildronate group experienced a decrease in aerobic performance parameters (VO_2max_, VO_2_ at Anaerobic Threshold, oxygen pulse, maximum load) by Day 15, whereas the placebo group did not. Mood index decreased significantly in the Mildronate group, while the placebo group’s mood index increased.	Non-random allocation (based on exercise test performance), creating severe baseline inequalities (Mildronate group had significantly higher baseline VO_2max_). High risk of sex confounding due to the performance-based allocation method. Short intervention duration (15 days).
Chainikov et al. (2015)	Uncontrolled single-arm interventional trial	25 male professional youth ice hockey players Mean age 18.5 ± 1.2 years	Significant increases in attention volume, attention switching, and mental productivity by Day 42. Positive trends observed in visual memory and memory after interference. Conversely, subjective “comfort” and mental activation decreased by Day 42 (the number of players reporting “High Comfort” dropped from 7 to 1).	No placebo or control group. Reliance on neuropsychological test batteries with limited international validation. Reliance on subjective, self-reported endpoints for health and psychoemotional status. Contradictory outcomes regarding psychoemotional status not fully reconciled by the authors.

### The athlete paradox: why use meldonium?

Despite the absence of robust, high-quality evidence for true ergogenic effects, Meldonium was consumed en masse by athletes prior to its 2016 prohibition. This paradox is largely explained by its historical legality, low cost, and long-standing cultural integration into Eastern European sports systems. For athletes who intentionally continued its use post-prohibition, accepting the severe risk of lengthy doping bans, the deeply ingrained perceived benefits of improved recovery and delayed fatigue likely outweighed the risk of detection. This phenomenon reflects a complex interplay between sports psychology, the powerful placebo effect, and theoretical pharmacology.

### Doping in sport and regulatory response

Meldonium's classification as a prohibited substance was catalyzed by evidence of widespread systemic use, most notably at the 2015 Baku European Games (61, 62). During the event, only 23 of 662 athletes' self-declared Meldonium use, yet anti-doping laboratories detected the drug in 66 of 762 samples, revealing substantial underreporting and a remarkably high true prevalence (61). Consequently, the decision to ban Meldonium was driven primarily by this alarming prevalence data and theoretical mechanistic concerns, rather than convincing human performance trials (38, 39).

The fallout from the ban was massive: between 2016 and 2024, Meldonium consistently ranked as the first or second most common Adverse Analytical Finding (AAF) in annual WADA reports (Table 2).

**Table 2:** WADA adverse analytical findings (AAFs) for meldonium from 2016 to 2024.

Year	Total AAFs	Global Testing Context & Notes
2016	515	The “Spike” Year: Meldonium was officially added to the WADA Prohibited List. Because of its long elimination half-life and widespread prior use, it caused a massive spike in global violations. Number one in the class “S.4 Hormone and Metabolic Modulators”
2017	79	The Drop-Off: A sharp decline as awareness of the ban grew and the drug washed out of athletes’ systems.
2018	111	Slight rebound/stabilization as testing methodologies improved globally. Number one in the class “S.4 Hormone and Metabolic Modulators”
2019	79	Resumed downward stabilization. Number two in the class “S.4 Hormone and Metabolic Modulators”
2020	26	Pandemic Impact: The number of positive tests artificially plummeted due to the COVID-19 pandemic, which caused a 46% drop in WADA’s overall global sample collections. Number two in the class “S.4 Hormone and Metabolic Modulators”
2021	70	Global testing began to normalize post-pandemic. Number one in the class “S.4 Hormone and Metabolic Modulators”
2022	70	Number one in the class “S.4 Hormone and Metabolic Modulators”
2023	77	Number one in the class “S.4 Hormone and Metabolic Modulators”
2024	63	Recent 2024 WADA data confirms that despite nearly a decade on the prohibited list, Meldonium violations remain persistent at baseline levels. Number two in the class “S.4 Hormone and Metabolic Modulators”

Data from WADA Anti-Doping Testing Figures 2016 to 2024.

Following an initial surge in positive tests, WADA acknowledged that Meldonium can persist in the body for unexpectedly prolonged periods, prompting guidance to treat low-level findings from before 1 March 2016 with caution (40, 61). Although case numbers have gradually declined, isolated violations continue. Ultimately, scientific consensus maintains that Meldonium's true ergogenic effects in humans remain unproven, and experts characterize its widespread reputation as a powerful performance enhancer as largely anecdotal (38, 39, 62).

### Proposed benefits vs. available evidence

#### Metabolic shifts

In animal models, Meldonium alters carnitine metabolism, decreases fatty-acid *β*-oxidation, and increases glucose utilization—changes that may be advantageous in hypoxic or ischemic conditions (11, 15, 63, 64). However, in the only available human study on trained athletes, this substrate shift appeared maladaptive: the Meldonium group showed a lower VO₂_max_ and workload alongside an increased RER, suggesting impaired—or at least not improved—maximal performance (59).

#### Aerobic capacity and lactate

Animal work reports reduced oxidative stress and sometimes lower lactate accumulation following Meldonium administration (11, 12, 17, 32, 39, 65). In contrast, Goloborodko et al. observed significantly lower O₂ pulse and higher VE/VO₂ with Meldonium compared with placebo, patterns consistent with reduced aerobic exercise capacity, and no between-group differences in blood lactate 5 min after maximal exercise (59).

#### Anaerobic capacity

In obese rats, Meldonium lowered blood lactate concentrations during high-intensity exercise (34). However, no human study has demonstrated improved anaerobic capacity or altered lactate kinetics attributable to Meldonium (39, 59).

#### Recovery and cardioprotection

Meldonium may shorten recovery after cardiac events and improve indices of cardiac function in patients with coronary disease or diabetes, as well as in animals exposed to high altitude or other stressors (18, 29, 64, 66–69). These findings underpin the interest in meldonium as a cardioprotective adjunct. However, to date, no published human study has rigorously shown improved post-exercise recovery, exercise-induced cardioprotection, or enhanced cardiac function in healthy athletes following meldonium intake.

Taken together, the mechanistic data have frequently been interpreted to suggest that Meldonium might potentiate training adaptation through AMPK/PPAR*δ*–PGC-1*α*–mediated mitochondrial biogenesis and eNOS-dependent improvements in perfusion. Yet, this remains highly speculative. The only controlled study in trained athletes (59) reports no improvement (and possible impairment) of maximal performance, and no trial has directly linked these molecular signaling changes to superior training outcomes in healthy, well-trained humans. While three clinical trials evaluating Mildronate® are currently registered on ClinicalTrials.gov (in China, Albania, Azerbaijan, and the Russian Federation), published results have not yet clarified its role in exercise performance or recovery.

### Mechanistic implications for endurance athletes

Inhibiting mitochondrial fatty-acid uptake acutely forces a greater reliance on glucose and glycogen during exercise. For endurance athletes, this can be distinctly disadvantageous: accelerated glycogen depletion may precipitate earlier fatigue and reduce sustained performance within a single session, particularly before any long-term adaptations can occur. While chronic exposure might partially normalize lipid oxidation via changes in gene expression and mitochondrial remodeling, the short-term effect is logically a decrease in exercise tolerance and potentially prolonged recovery. These substrate-use trade-offs highlight the critical need for human studies that address both performance and long-term safety outcomes (including hepatic and renal function) when using fatty-acid oxidation inhibitors.

## Conclusion and future research

Despite the widespread consumption of meldonium by elite athletes leading up to its 2016 WADA prohibition, this review reveals a notable absence of high-quality clinical evidence supporting its ergogenic efficacy. To date, only a single, methodologically limited, placebo-controlled trial has evaluated its effects on maximal physical performance in healthy athletes, and its results actually point toward negative trends in both physical and mental performance (59).

Consequently, Meldonium's international reputation as a powerful performance enhancer appears to be built primarily on anecdotal use, theoretical biochemical mechanisms, and the inappropriate extrapolation of data from compromised animal models and ischemic patients. These findings actively challenge the rationale for meldonium as a performance-enhancing agent and raise unresolved concerns about its psychological tolerability, possible interference with physiological adaptation, and long-term safety profile (20).

In light of these methodological limitations, it is premature to definitively classify meldonium as either an effective or ineffective doping agent. Its continued misuse appears to rest more on tradition than on scientific evidence, supporting the view that its ergogenic potential remains fundamentally unproven (62). Resolving this discrepancy between widespread anti-doping policy and the current hierarchy of evidence will require rigorous, randomized, and placebo-controlled international trials, though the ethical and practical realities of conducting such studies in elite sports populations remain a significant barrier.
